# Ultrafine Airborne Particle Deposition and Clearance in an Avian Lung under Ecologically Relevant Conditions

**DOI:** 10.1021/acs.est.6c05957

**Published:** 2026-08-24

**Authors:** Susana Garcia Dominguez, Linnéa Jönsson, Andreas Nord, Calle Preger, Maria E. Messing, Caroline Isaksson, Jenny Rissler

**Affiliations:** † Department of Biology, 5193 Lund University, 223 62 Lund, Sweden; ‡ Solid State Physics, Department of Physics, Lund University, 221 00 Lund, Sweden; § NanoLund, Lund University, 221 00 Lund, Sweden; ∥ Swedish Centre for Impacts of Climate Extremes (climes), Lund University, 221 00 Lund, Sweden; ⊥ Ergonomics and Aerosol Technology, Department of Design Sciences, Lund University, 221 00 Lund, Sweden; # MAX IV Laboratory, Lund University, P.O. Box 118, Lund 221 00, Sweden; ¶ Quantum Device Laboratory, Department of Microtechnology and Nanoscience, Chalmers University of Technology, 412 96 Gothenburg, Sweden

**Keywords:** birds, inhalation exposure, lung deposition, particulate matter, avian respiratory system

## Abstract

Air pollutants, such as particulate matter, are known to contribute to disease in humans, but their impact on wildlife remains understudied. A contributing factor to this health impact is the fraction of inhaled particles depositing in the respiratory system. Compared to humans, birds are equipped with a very different respiratory system, which may render them more or less susceptible to air pollution. Although evidence for adverse pollution effects in birds has emerged, little is known about particle dynamics inside the avian lungs. To elucidate this, we exposed zebra finches (*Taeniopygia guttata*) to ambient-like ultrafine model particles under controlled conditions and quantified deposition in the lungs and, in a smaller subset, also the heart, liver and red blood cells. Birds were exposed to 50 or 100 nm particles at air temperatures of 5 or 25 °C, all of which fall within environmentally relevant exposure conditions. Lung deposition was highest for the smallest particles, in line with the size dependence of particle diffusion rate. While deposited fraction in the lung remained unaffected, deposited dose rate (DR) increased at the lower temperature, indicating that the effect was driven solely by elevated minute ventilation (oxygen consumption). Particle clearance measurements 2 weeks after exposure revealed that 44% of the initially deposited 100 nm particles remained in the avian lungs. We found no evidence that particles translocated to the heart, liver or red blood cells in the analyzed subsets. These findings provide an ecologically relevant foundation for understanding particle exposure dynamics in an avian model and support future work assessing health effects of particulate pollution in birds.

## Graphical abstract

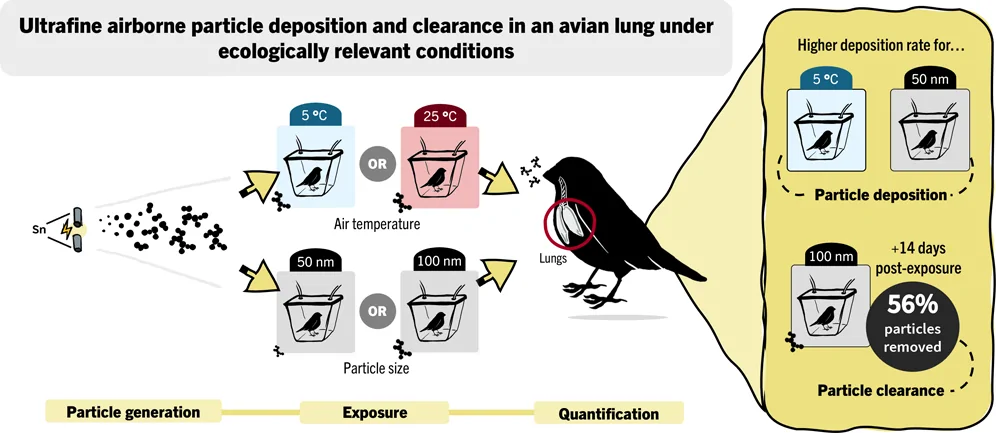

## Introduction

The dramatic increase in industrialization and urbanization of the last century has significantly contributed to today’s environmental challenges, such as climate change, habitat loss and air pollution.^1–3^ These environmental problems are leading to a global decline in biodiversity, ecosystem degradation, and poorer health in both wildlife and humans.^4–7^ In urban environments, traffic and domestic heating (i.e., biomass burning), as well as industrial processes (e.g., power plants, chemical processing, smelting and refining), release large quantities of particulate matter (PM) into the air,^8^ which can be inhaled and deposited in the respiratory system. Human exposure to PM is associated with a wide range of cardiovascular and respiratory diseases, cancer and other dysfunctions.^9,10^ However, outside the livestock literature (e.g., refs 11, and 12 ), much less is known about the impact of PM exposure in other vertebrates (but see refs 13–15 ).

In the human lungs, coarse particles (aerodynamic diameter, *d*_ae_ > 2.5 μm) mostly deposit in the upper airways, where they are cleared from the system by the mucociliary barrier.^16^ In contrast, fine (*d*_ae_ ≤ 2.5 μm) and ultrafine (*d*_ae_ ≤ 0.1 μm) particles can travel further into the lungs. Upon reaching peripheral lung regions, fine and ultrafine particles tend to deposit efficiently and are cleared slowly.^17^ Combined with their high number concentrations and large specific surface area (defined as surface area per mass unit; refs 18, and 19 ), these properties are thought to contribute to the stronger associations observed between ultrafine particles and adverse health outcomes compared to coarse particles.^20–22^ Ultrafine particles can also cross the lung barrier and reach the bloodstream, directly via the capillary beds within the alveolar wall or through the lymphatic system, potentially affecting extrapulmonary organs.^23^ Yet, the dynamics of inhaled particle deposition and clearance are largely unknown in birds, whose respiratory system is fundamentally different from that of mammals.

Birds possess a highly efficient respiratory system relative to similarly sized mammals,^24^ characterized by unidirectional airflow and cross-current gas exchange. This adaptation for high aerobic capacity enhances oxygen uptake, but may also increase exposure of pulmonary tissues to particulate pollutants.^14^ In addition, the blood-gas barrier in birds is thinner than in mammals, allowing for higher gas diffusion rates, and the respiratory surface area is larger.^25^ Unlike mammals, inhaled air remains in the respiratory system for two full respiratory cycles before being exhaled, contributing to longer residence times of particles in the avian system.^25,26^ Together, these features may lead to different deposited fractions in birds, with potential implications for their susceptibility to air pollution.^14^ Particle deposition in the lungs may also increase during periods of high metabolic demand, such as wintertime, when elevated ventilation rates are needed to meet the higher metabolic heat production required to maintain body temperature.^27^ The extent to which these metabolic demands influence particle deposition in birds remains untested.^15,19,66,67^

Few avian studies have examined deposition of airborne particles, with the existing research mainly using radiolabeled or fluorescent particles in the domestic fowl.^28–30^ These studies show that deposition in the lungs tends to decrease with increasing particle size (0.1–7 μm; 1–20 μm),^28,30^ and that particle clearance appears to be biphasic, with a faster initial phase followed by a slower one.^29^ However, part of the previous work on birds has been conducted on anaesthetized subjects, which may not reflect deposition under natural conditions as anesthesia alters avian respiratory dynamics^31,32^ and, potentially, particle deposition patterns. Additionally, deposition of ultrafine particles has barely been studied in birds.^28^ This is critical, as many ambient air pollution particles fall within the ultrafine size regime, including diesel soot particles and secondary aerosols formed through particle nucleation in the atmosphere, which typically peak at approximately 50–100 nm.^33–35^ Thus, how environmentally relevant particles deposit and are cleared in birds, especially in nondomestic species, is still an open question.

Here, we address this research gap by quantifying the deposition of ultrafine particles in the avian lungs. We exposed zebra finches (*Taeniopygia guttata*), an avian model species in many biological fields,^36^ to airborne monodisperse aggregate model Sn particles (50 or 100 nm in diameter) with physical properties similar to ambient ultrafine particles, and for 2 h. We determined deposited dose rate (i.e., deposited dose per hour and per particle mass concentration in the inhaled air) and estimated particle deposition efficiency, defined as the ratio between the particles depositing in a given region of the respiratory system relative to those reaching that region^37^—in our case, the avian lung. This has proven to be an important measure to predict particle exposure and health implications in humans.^38^ To test the role of metabolic rate in deposition, we exposed individuals to either a cold (5 °C) or a mildly warm air temperature (25 °C). Because diffusion is the major particle deposition mechanism for particles in this size regime,^39^ we predicted higher lung deposition rates for birds exposed to the smaller particles. Based on the expectation that deposition is temperature dependent, with metabolic rates (and, thus, breathing) increasing at the cold air temperature, we predicted higher lung deposition rates at the cold air temperature.

We also assessed the clearance of deposited particles from the avian lungs over a two-week postexposure period, and investigated whether particles reached the circulation (i.e., red blood cells), or extrapulmonary tissues (i.e., heart and liver) after exposure. Although the particles in the study were selected as model particles to represent the physical properties of air pollution, we complemented these measures by including an exploratory evaluation of physiological effects of short-term particle exposure. We measured (1) oxidative stress levels immediately after exposure, and (2) exposure-related changes in metabolic rate. As oxidative stress is related to the pathology of many illnesses,^40^ these measurements could offer insight into potential short-term systemic responses to particle exposure. As a whole, this work offers a detailed quantification of ultrafine particle deposition and clearance in an avian model, along with an exploratory evaluation of short-term physiological responses to particle exposure.

## Results

We exposed 60 healthy zebra finches to one of five treatments (overview in Figure 1): 50 or 100 nm particle aggregates at 25 °C (*n*_50_ = 10/*n*_100_ = 20), particle-free air at 25 °C (*n* = 10), 100 nm particle aggregates at 5 °C (*n* = 10), or particle-free air at 5 °C (*n* = 10). Fifty individuals were euthanized right after exposure to quantify particle deposition in the lungs, while ten birds from the group exposed to 100 nm particles at 25 °C were euthanized 2 weeks later to assess particle clearance.

**Figure 1:**
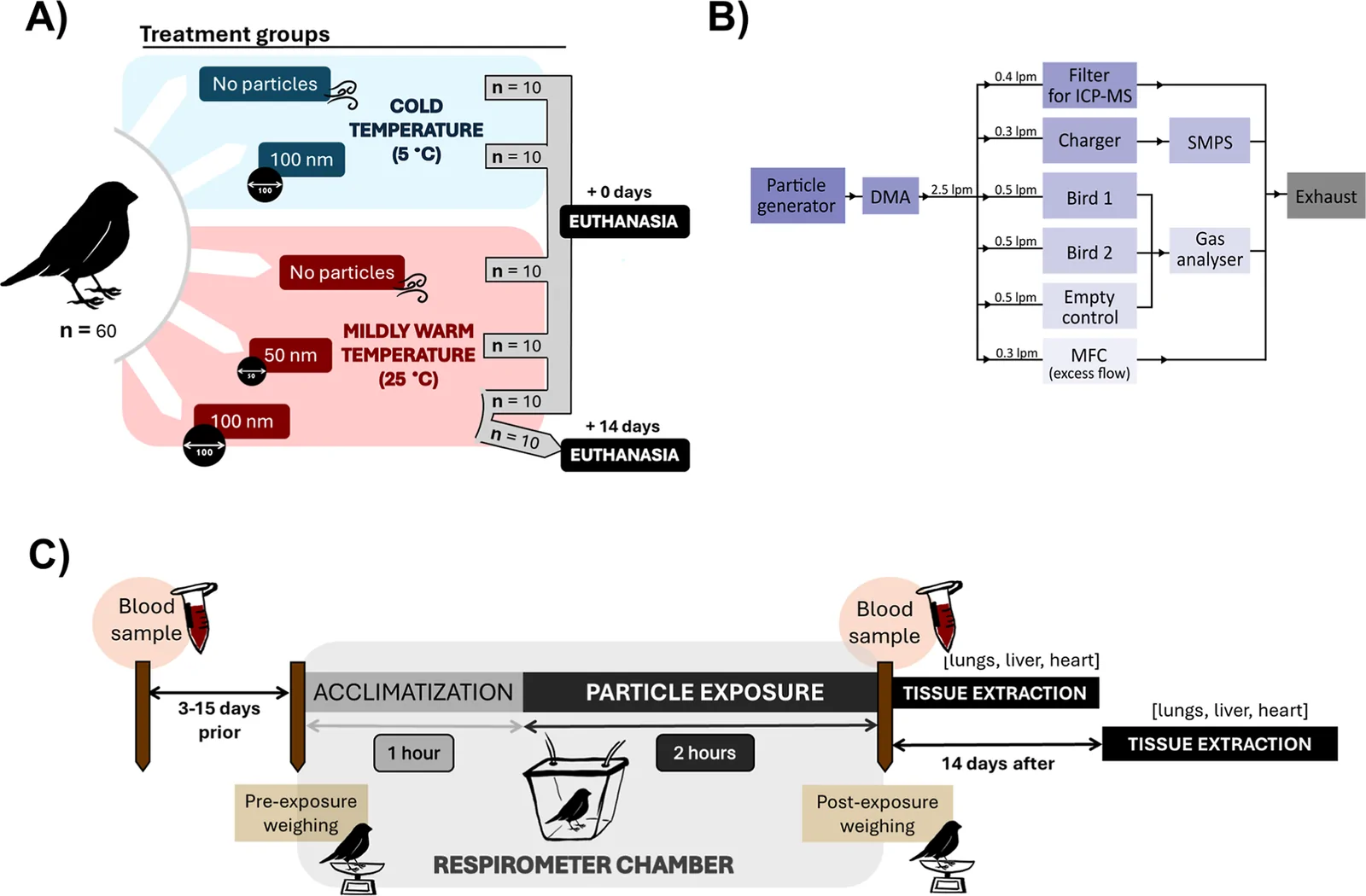
Experimental design and setup overview. (A) Experimental treatments in which individuals were exposed to either particle-free air or 100 nm (in mobility diameter) particles at cold temperature (5 °C), or particle-free air, 100 nm particles or 50 nm particles at warm temperature (25 °C). Tissue extraction took place right after exposure or 14 days after. Sample sizes (*n*) included in the diagram. (B) Instrumental setup for particle generation and characterization. The flow of quasi monodisperse particles left the generator (2.5 L·min^–1^) and split into six sub flows: (1) to filters for determining the mass exposure by ICP–MS, (2) to online monitoring of the number size distribution (the SMPS), (3,4) into the chambers where the birds were placed, (5) to empty chamber for reference. Note that all flowlines had a filter to ensure removal of all particles before going to the gas analyzer. The excess flow (6) was monitored by a mass flow meter but not steered. (C) Experimental timeline including blood sampling and body mass measurements before and after exposure, exposure with acclimatization period, and tissue extraction of lungs, liver and heart for quantification of particle deposition and clearance. Abbreviations: SMPS = scanning mobility particle sizer, DMA = differential mobility analyzer, ICP–MS = inductively coupled plasma mass spectrometry.

### Particle Size and Morphology

The generated particles are aggregates composed of several primary particles (Figure 2A). For these nonspherical particles, the term “particle diameter/size” hereafter refers to the aggregate mobility diameter, defined as the diameter equivalent to that of a sphere with the same diffusion rate.^41^ The geometric mean of the main peak in the average mass size distributions in each exposure type was 49.6 ± 0.8 nm and 103.7 ± 1.5 nm, estimated from the measured number size distributions (nominally 50 and 100 nm; Figure 2B), where the given standard deviation (±) corresponds to the variation between exposure occasions. The particles consist of smaller, primary particles stuck together, forming larger particle aggregates (Figure 2A). The average primary particle diameter was determined to be 4.4 ± 0.8 nm (mean ± SD, 50 nm sample) and 4.3 ± 0.9 nm (100 nm sample).

**Figure 2:**
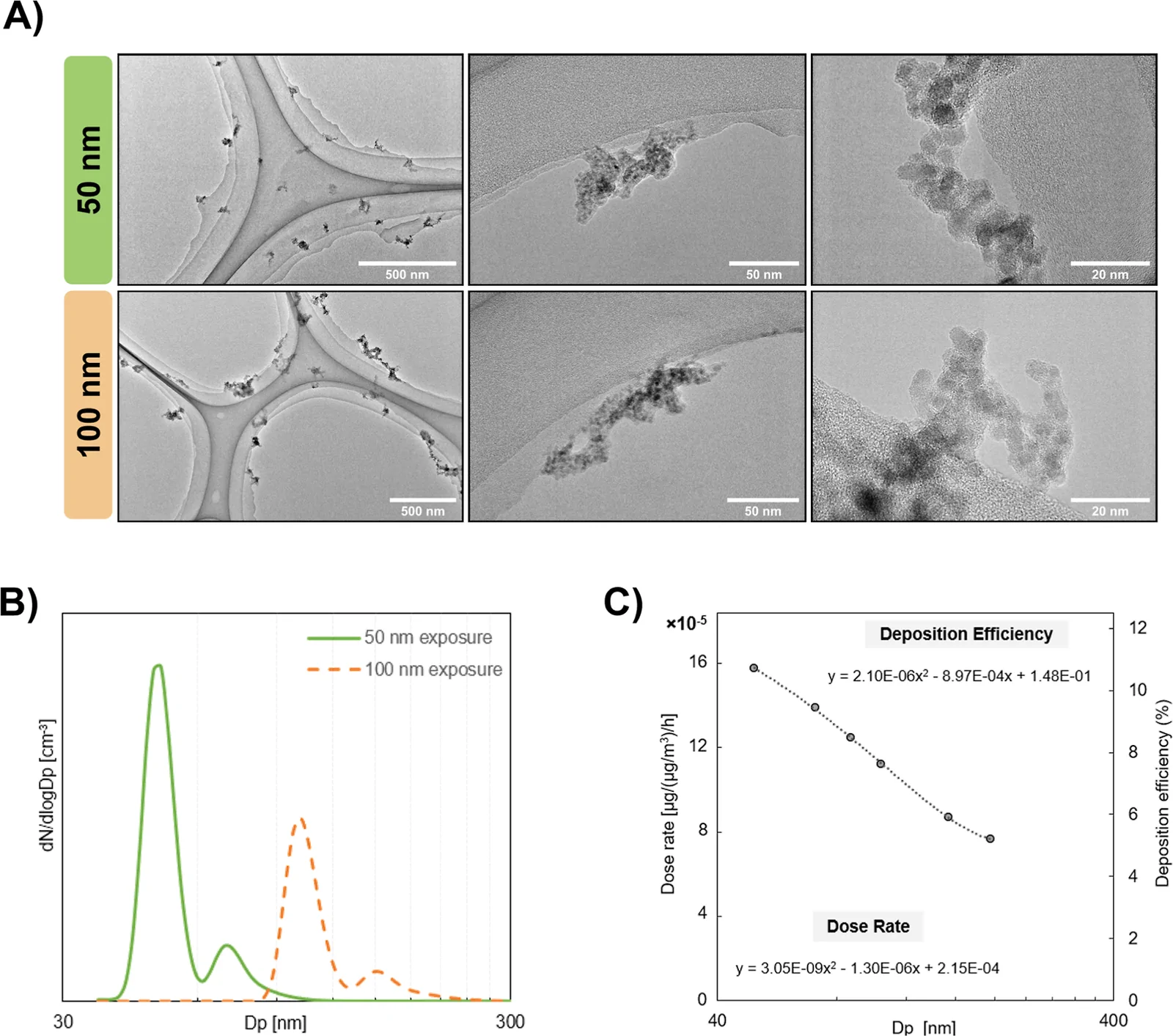
(A) Sn oxide particles with mobility diameters of 50 nm (top) and 100 nm on filters (bottom). The average primary particle diameter was ∼4.4 nm and ∼4.3 nm, respectively. Images were taken using a transmission electron microscope (TEM, JEOL 3000F) at the national center for high resolution electron microscopy (nCHREM) in Lund, Sweden. (B) Number size distributions during exposures of 50 and 100 nm particles (50 nm, solid line and 100 nm, dashed line). In the size distributions, larger particles are also observed due to doubly charged particles passing the differential mobility analyzer (DMA). For the 50 and 104 nm particles, these appear at 71 and 153 nm, respectively, corresponding on average to 30% and 34% of the total particle mass. (C) Calculated size dependent dose rates from the study (left axis) with corresponding deposition efficiency (right axis). The equations describe the size dependent dose rate and deposition efficiency, respectively. Note that the *x*-axes in both (B) and (C) are shown on a logarithmic scale. Abbreviations: *D*_p_ = particle size.

### Particle Mass and Number Concentrations

The particle exposure levels (mass and number) and size were monitored in parallel to each exposure session (details in Materials and Methods). At 25 °C, the average particle mass concentration in the air was, for 50 nm particles, 64 ± 4 μg·m^–3^ (mean ± SD, corresponding to 9.0 × 10^5^ particles·cm^–3^), and for 100 nm particles, 165 ± 42 μg·m^–3^ (5.7 × 10^5^ particles·cm^–3^, Table 1). At 5 °C, the 100 nm particle mass concentration was slightly lower, 156 ± 31 μg·m^–3^ (5.2 × 10^5^ particles·cm^–3^). During the exposures for determining particle clearance, the mass concentrations were 222 ± 18 μg·m^–3^ (6.8 × 10^5^ particles·cm^–3^, Table 1). The varying particle concentrations during exposures affected the absolute dose to the lungs, but this was accounted for by normalizing deposited dose in lungs to exposure levels (see “Deposited Dose Rate (DR) in the Lungs” subsection).

**Table 1:** Average Particle Mass in Air and Corresponding Number Concentrations per Treatment Group^a^

treatment group	average total particle concentration in air (μg·m^–3^)	corresponding total number concentration (·10^5^ particles per cm^–3^)
clearance period of 0 days		
100 nm particles, 5 °C	156 (103) ± 31 (17)	5.17 ± 0.93
100 nm particles, 25 °C	165 (108) ± 42 (24)	5.73 ± 1.35
50 nm particles, 25 °C	64 (45) ± 4 (3)	8.97 ± 0.30
clearance period of 14 days		
100 nm particles, 25 °C	222 (145) ± 18 (10)	6.75 ± 0.43

a Total concentrations of particles (mean ± SD) shown here. For mass concentrations, single charged particle values are also provided in brackets.

### Deposited Particle Dose in the Lungs

Even though the mass concentrations of Sn in air during the 2 h exposures of 50 nm particles were less than half of the mass concentration of the 100 nm particles at 25 °C, the dose to the lungs was just slightly lower than for the 100 nm particles. The total deposited particle mass in the lungs, expressed as Sn mass, was 0.019 ± 0.001 μg (mean ± SE, *n* = 10) and 0.025 ± 0.002 μg (*n* = 10) for exposures of 50 and 100 nm particles, respectively. At 5 °C, the dose for 100 nm particles was the highest, 0.040 ± 0.004 μg (*n* = 10), despite the similar Sn mass concentrations in the air at both temperatures. In individuals used to assess particle clearance, the particle dose remaining in the lungs 2 weeks after exposure was 0.019 ± 0.002 μg (*n* = 10). The slightly higher concentrations in the air during these experiments were adjusted for in the interpretation of the results. Control individuals (i.e., exposed to particle-free air, *n* = 20) had a mean deposited particle dose in the lungs of 0.005 ± (<0.001) μg (*n* = 20), which was much lower than all the particle-exposed individuals (Mann–Whitney *U* test, *U* = 0, *p* < 0.001, *n* = 60). Descriptive statistics can be found in Supporting Information (Table S2).

### Particle Translocation to Circulation or to Extrapulmonary Organs

In the red blood cells of a subset of individuals (*n* = 14), Sn levels were no different than a blank sample. In the heart and liver of another subset of individuals (*n* = 14), deposited Sn dose of 100 nm particles at 25 °C was below detection limits, (i.e., <0.005 μg of Sn·g^–1^ avian tissue), regardless of clearance period (i.e., 0 or 14 days).

### Deposited Dose Rate (DR) in the Lungs

The dose to the lungs is dependent on exposure time, inhaled concentration and deposited fraction. To have a more general measure not affected by slight variations in exposure levels, we use the concept of “deposited dose rate” (DR), which is a measure of the particle lung deposition normalized to particle exposure (i.e., measured lung deposited particle mass divided by exposure duration and particle mass concentration in the air, see eq 3 in Materials and Methods).

At 25 °C, DR was 90% higher in individuals exposed to 50 nm particles (marginal means ± SE: 1.56 × 10^–4^ ± 6.38 × 10^–6^ μg·h^–1^ per μg·m^–3^ in air) than in those exposed to 100 nm particles (0.82 × 10^–4^ ± 6.38 × 10^–6^ μg·h^–1^ per μg·m^–3^ in air, *F*_1,16_ = 65.86, *p* < 0.001; Figure 3A, Table S3). Neither sex nor body mass had a statistically significant effect on DR (*p* > 0.28, Table S3).

**Figure 3:**
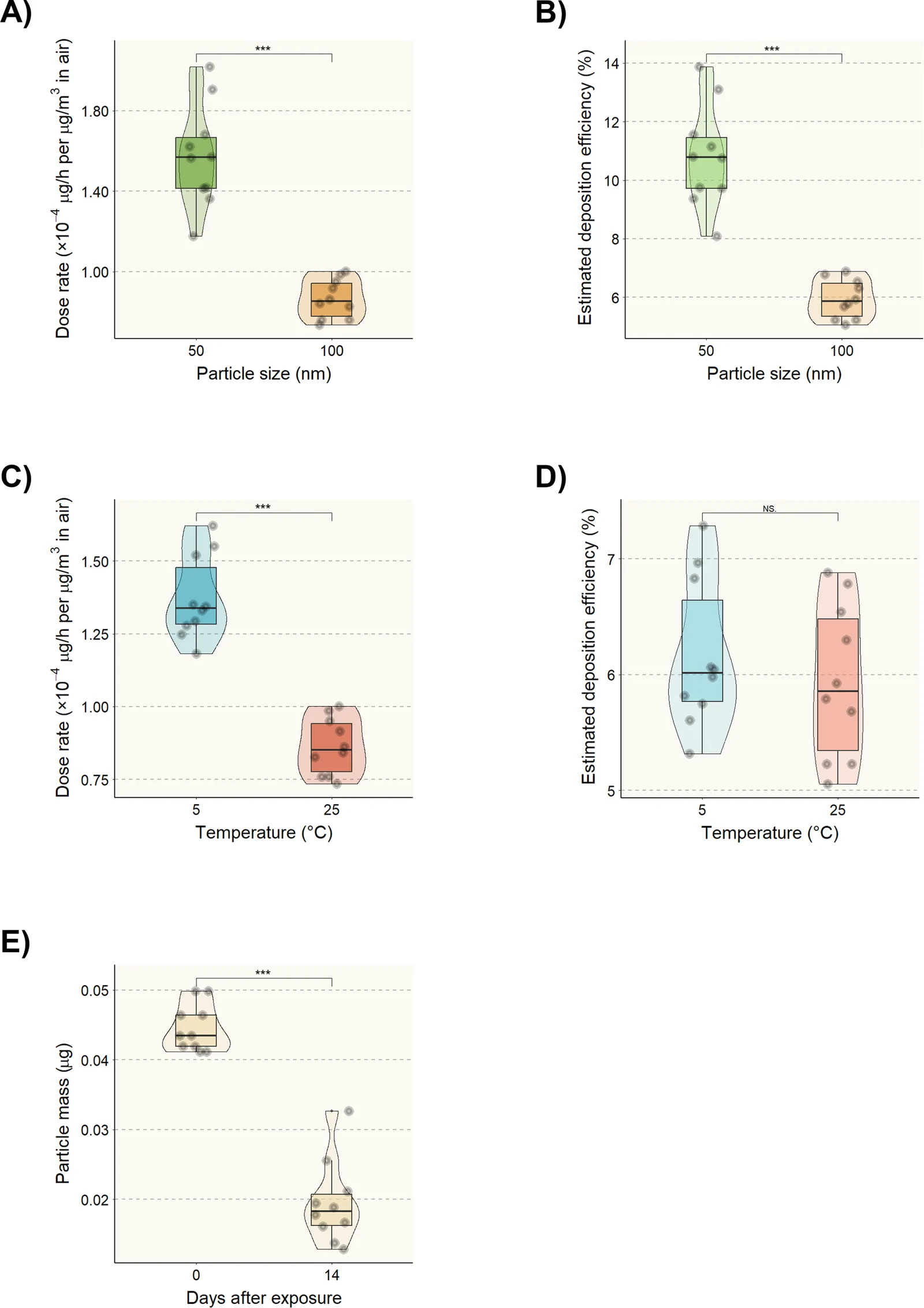
Treatment effects on particle deposition and clearance in zebra finches. Effect of particle size on deposited particle dose rate, derived from direct measurements of particle mass in the lungs (A), and estimated particle deposition efficiency (B) at 25 °C. Effect of ambient temperature on deposited particle dose rate, derived from direct measurements of particle mass in the lungs (C), and estimated particle deposition efficiency (D) of 100 nm particles. (E) Estimated deposited particle mass in the lungs right after exposure (0 days) and measured remaining deposited mass 2 weeks after (14 days) of 100 nm particles. Data points are shown as large dark gray dots. (***) indicates *P* < 0.001, and “NS.” indicates *P* > 0.05.

Additionally, based on the particle size exposure data of this study, we fitted a second order polynomial (*R* ^2^ = 1.00) to the size dependent DR, shown below (eq 1). This resulting equation can be used to calculate DR of other particle sizes within the size range of this study (i.e., 50–200 nm, Figure 2C):

1 $$DR={3.05\times 10^{-9}\times D}_{p}^{2}-{1.30\times 10^{-6}\times D}_{p}^{1}+2.15\times 10^{-4}$$

*D*_p_ = particle size.

DR was 59% higher in the cold climate (5 °C, marginal means ± SE: 1.37 × 10^–4^ ± 3.87 × 10^–6^ μg·h^–1^ per μg·m^–3^ in air) than in the mildly warm climate (25 °C, marginal means ± SE: 0.86 × 10^–4^ ± 3.87 × 10^–6^ μg·h^–1^ per μg·m^–3^ in air, *F*_1,16_ = 86.20, *p* < 0.001; Figure 3C, Table S3). Neither sex nor body mass had a statistically significant effect on DR (*p* > 0.18, Table S3).

### Estimation of Particle Deposition Efficiency (DE)

Another, more general measure of particle lung deposition, is deposited particle fraction or deposition efficiency (DE). DE is a measure of the risk that a particle, once inhaled, will be deposited in the lung and is given as % (eq 4 in Materials and Methods).

DE was 76% higher for 50 nm particles (marginal means ± SE; 10.69% ± 0.44) than for 100 nm particles (6.06% ± 0.44; *F*_1,16_ = 65.86, *p* < 0.001, Figure 3B, Table S3). DE was not affected by body mass or sex at a statistically significant level (*p* > 0.05, Table S3).

As for DR, we fitted a second order polynomial (*R* ^2^ = 1.00) to the size dependent DE, which can be used to calculate DE of other particle sizes (Figure 2C, eq 2):

2 $$DE={2.10\times 10^{-6}\times D}_{p}^{2}-{8.97\times 10^{-4}\times D}_{p}^{1}+1.48\times 10^{-1}$$

*D*_p_ = particle size.

There was no difference in DE between temperatures (*F*_1,16_ = 0.59, *p* = 0.45, Figure 3D, Table S3), indicating that the difference in dose between temperatures is caused by an alteration in ventilation rate. Thus, the dose increases at lower temperatures because the volume of inhaled air rises, whereas the probability that an inhaled particle is deposited remains independent of temperature. DE was not affected by body mass nor sex at a statistically significant level (*p* > 0.15; Table S3).

### Simplified Theoretical Calculations of Particle Deposition

A first-order assessment of particle deposition in the avian respiratory system was performed using fundamental aerosol physics and an idealized flow-through representation of the avian respiratory tract.

As hypothesized, the observed size-dependent DEs in the lungs could be reproduced by the simplified model assuming diffusion as the sole deposition mechanism. To match the experimental DEs, the product of the number of parabronchioles and an effective aerodynamic parabronchial length was fitted to the data. Because diffusion-driven deposition in a flow-through system depends on the factor between these parameters, they cannot be independently resolved. The fitted ratio was approximately 1.5 [m·number of parabronchioles]. Assuming 300 parabronchioles (number reported by ref 42), the effective aerodynamic parabronchial length is 5 mm.

As only the lungs were collected and analyzed, the measured DEs represent deposition within the lungs and exclude deposition in the trachea and air sacs. However, agreement between modeled and measured DEs was achieved only when an additional deposition compartment representing particle losses upstream of the lungs was included. The calculations therefore indicate substantial deposition in the trachea and air sacs before particles reach the pulmonary region, consistent with expectations based on aerosol physics and avian respiratory anatomy.

### Estimation of Particle Clearance from the Lungs

We measured the dose remaining in the lungs of individuals euthanized 2 weeks after exposure. Clearance was determined by comparing this remaining dose to the mean dose of individuals euthanized immediately after exposure to same size and temperature (100 nm at 25 °C), while accounting for the slight difference in particle concentration in air during exposure (see Materials and Methods for details). Estimated particle clearance was 56%, meaning that 56% of the estimated initially deposited particle mass in these birds had been removed, and 44% remained in the lung after 2 weeks (*t*_9_ = −10.44, *p* < 0.001; *n* = 10, Figure 3E).

### Effect of Particle Exposure on Metabolic Rate

We tested the effect of particle exposure on metabolic rate (in *W*) separately per temperature treatment. The interaction between treatment and measurement time point on metabolic rate was not significant either at the cold (*F*_1,78_ = 0.09, *p* = 0.76, Table S4) or at the warmer temperature (*F*_2,157_ = 1.40, *p* = 0.25, Table S4).

In the cold temperature, metabolic rate increased from the acclimatization hour to the end of the 2 h exposure (*F*_1,79_ = 12.32, *p* < 0.001), resulting in an estimated increase in minute volume ventilation rate. This increase happened regardless of whether individuals were exposed to particles or not (*F*_1,17_ = 1.15, *p* = 0.30, Table S4). Body mass had no effect on metabolic rate (*p* > 0.15, Table S4). In the warm temperature, metabolic rate was not significantly affected by any predictor variable (*p* > 0.12; Table S4).

### Effect of Particle Exposure on Oxidative Status Biomarkers in Plasma

Nonenzymatic antioxidant capacity (mM HClO neutralized; *F*_4,43.83_ = 0.30, *p* = 0.88) and oxidative damage levels measured as hydroperoxides (mM H_2_O_2_, *F*_4,39.88_ = 1.82, *p* = 0.14) were not affected in any of the exposure treatments (particle size or air temperature, Table S5) at these relatively low doses. Postexposure levels were tightly linked to pre-exposure baseline levels of the same individuals for both biomarkers (nonenzymatic antioxidant capacity: *F*_1, 44.85_ = 38.46, *p* < 0.001; oxidative damage: *F*_1, 40.90_ = 11.70, *p* = 0.001; Table S5). Sex had no statistically significant effect on either of the biomarkers (*p* > 0.08, Table S5).

## Discussion

Using a novel experimental approach, we found that the deposition rate was size dependent, showing a higher deposition rate for 50 nm particles compared to 100 nm in a zebra finch model. The resulting deposition efficiency (i.e., % deposited particles of those inhaled) was approximately 11% of inhaled 50 nm particles compared to 6% for 100 nm particles. This size dependence effect was expected as ultrafine particles deposit primarily by diffusion (their Brownian motion), with smaller particles having a higher diffusion coefficient and, consequently, a greater likelihood of reaching and being deposited on airway walls.

Our simplified theoretical calculations of particle deposition support this interpretation, furthermore showing that a proportion of the inhaled particles are also deposited in the trachea and air before reaching the pulmonary region, which is to be expected. This size dependence is also consistent with studies in humans, despite such studies typically reporting higher deposited fractions in the deep lungs. For comparison, the estimated average particle deposition efficiency in the alveolar region of the human lung is approximately 25–30% for 50 nm particles, and 15–20% for 100 nm particles, based on results from experimental studies^38^ combined with modeled alveolar deposition fractions.^43^ To date, however, no generally applicable deposition models have been developed for the avian respiratory system. The higher deposited fraction in humans likely reflects fundamental differences in respiratory anatomy. A recent three-dimensional reconstruction of the zebra finch pulmonary anatomy by ref 42, together with earlier anatomical studies,^25,26^ also emphasize these differences. Unlike birds, which are equipped with a mostly unidirectional, flow-through breathing system,^26,44^ humans rely on bidirectional, tidal breathing. The human airway geometry favors lower airflow velocity and longer particle residence times in the alveolar region, contributing to higher deposition by diffusion.^45^ Although this difference does not necessarily imply that birds are less affected by ultrafine particles than humans, it underscores the need for a system-specific understanding of particle deposition patterns outside mammalian models, including deposition models specifically tailored to avian respiratory function.

We also observed that particle deposition rate, but not deposition efficiency, was higher in the cold condition (5 °C compared to 25 °C). These findings match our initial hypotheses that the higher ventilation rate needed to increase metabolic rate in this condition will result in the higher dose rates, while the probability of deposition once a particle is inhaled is not temperature dependent. This can be compared with observations in humans, where deposition rates increase during exercise—a period of elevated metabolic demand—while deposited fraction remains unchanged.^46^ Birds may therefore receive a higher particle dose during energetically demanding periods, such as cold weather, or sustained flight.^47^ In many temperate areas, ambient particle concentrations in urban areas tend to be higher in the cold season, due to increased domestic heating emissions (e.g., biomass burning), traffic (e.g., engine start efficiency)^48^ and meteorological conditions (e.g., temperature inversions, lower boundary layer height) that might trap pollutants near the ground.^49–52^ Thus, environmental and physiological factors may act synergistically, increasing particle exposure in birds during the cold season. Even in the absence of higher ambient particle concentrations or changes in deposition efficiency, our findings suggest that increases in metabolic demand result in higher deposition rates, highlighting that particle dose cannot be inferred from exposure levels alone.

Under our experimental settings, we estimated that zebra finches had cleared about 56% of the deposited 100 nm particles 2 weeks after exposure. This is higher than what has been reported in humans. For instance, only 4.3% of labeled ultrafine particles (58–124 nm) were cleared from human lungs 1 week after particle administration.^53^ In chickens exposed to fine and coarse radiolabeled particles, 46% of the initially deposited mass was cleared within the hour, and 36 h after exposure, this number increased to 64%.^29^ However, the percentage of cleared particles did not significantly differ between 12 and 36 h after exposure, suggesting that the larger bulk of particle clearance happens early. Studies employing radiolabeled particles have however been subject to debate due to concerns regarding the stability of the radiolabel, particularly whether it remains firmly attached to the particles upon deposition.^54^ In contrast, our approach directly quantified the chemical components of the particles, eliminating the uncertainty associated with the risk of label detachment. Yet, although we estimated substantial particle clearance within 2 weeks after exposure, the timeline for full particle clearance in the avian lungs remains unknown. In highly polluted environments, clearance mechanisms (e.g., particle removal by macrophages)^55^ could become overwhelmed by chronic particle deposition, potentially leading to increased particle accumulation over time. In human studies, while half of the deposited particles in the bronchiolar region are removed within a day, clearance of those deposited in the alveoli can extend over several months.^17,56^ As temporal patterns in birds seem to differ from those of mammals, there is a need for longer term investigations of clearance in the avian respiratory system.

We did not detect any Sn particles in the heart, liver or red blood cells, either immediately after exposure or 2 weeks later, which may indicate that inhaled particles never reached those organs in the present study. Similarly, but in chicken embryos instead of adults, most metal nanoparticles larger than 30 nm remained in the lungs for the hour following tracheal injection.^57^ However, at least in our study, it is also possible that particle levels were simply below the detection limit of our method of analysis (inductively coupled plasma mass spectrometry, ICP–MS) outside the lungs. This dilution effect across the body is especially plausible for individuals sampled 2 weeks after exposure, as part of the deposited mass may have been cleared out through other routes. In chickens, the gastrointestinal tract appears to be the main clearance pathway of coarse and fine particles after deposition, with particle levels increasing in this region while decreasing in the lungs in the hours following exposure.^29^ Although we did not measure concentrations in the gastrointestinal tract, a similar issue with detectability could have occurred, especially if clearance happened via several routes. To clarify such dynamics, deposition and clearance rates beyond the avian lungs need to be investigated in more detail across a broader tissue range.

Our main goal was to quantify the deposition of ultrafine particles in the avian lungs, with the model particles being therefore selected based on their physical properties, which are primarily responsible for determining deposition rates. Although their chemical composition was not chosen to represent ambient particles, the exposure could be argued relevant for work-place related settings. Therefore, we also evaluated whether exposure to SnO_*x*_ particles induced any physiological effects. We found no evidence that metabolic rate or the studied oxidative status biomarkers (i.e., nonenzymatic antioxidant capacity and oxidative damage via reactive oxygen metabolites) were affected by particle exposure. Inhalation studies in rodents have shown that oxidative stress occurs after exposure to ultrafine particles (e.g., carbon black, metals), but at much higher exposure concentrations (at the mg·m^–3^ range) and longer durations (e.g., refs 58, and 59 ). Thus, the dose and duration of our exposures were relatively low and/or too short to significantly disrupt avian physiology. Additionally, other factors such as particle composition and study tissue may have also played a role. Tin oxide is considered a relatively less reactive metal compared to other typically used materials (e.g., silver or copper), and local oxidative stress may have occurred in the lungs but remained undetectable at the systemic level. As particles were not detected in circulation, and we only measured oxidative stress in plasma, the lack of effect is not surprising.

Overall, our study presents novel data on ambient-like ultrafine particle deposition, retention, and clearance in the avian lungs. We also show how a lower air temperature results in higher particle deposition rate due to increased metabolic rate, and briefly explored the effect of our short-term exposures on avian physiology using oxidative stress biomarkers. Given the scarcity of air pollution studies integrating physiological measurements with aerosol physics in animals, our findings offer valuable insights into particle dynamics in a respiratory system fundamentally different from that of mammals. Ultimately, such understanding can help evaluate the ecological and health consequences of particulate pollution in birds, especially those in highly urbanized or industrialized environments, where pollutant exposure is the highest.

## Materials and Methods

### Ethics Statement

All animal procedures were carried out following an ethical permit approved by the Malmö-Lund Animal Ethics Committee (number 5.8.18-10371/2022).

### Animal Subjects

60 zebra finches (30 males and 30 females, aged between 5 and 6 years old) were selected from a captive population of known pedigree. Individuals in this population lived in same-sex outdoor aviaries (*L* × *W* × *H*: 3.5 × 3.7 × 2.3 m) at Stensoffa Ecological Field Station (Sweden) and always had access to food (mixed seed diet, Exoten, Benelux, Kinlys Group, Belgium), water and cuttlebone pieces to boost their calcium intake. Test individuals were moved from the outdoor aviaries to same-sex indoor aviaries (2 × 2 × 2.2 m) a week prior to the start of the experiment. The day before their exposure session, 6 individuals were transferred to same-sex cages (97 × 52 × 83 cm) in the animal facilities of the Biology Department. For both the indoor aviaries and cages, ambient temperature was set at 25 °C, and the light schedule was similar to the natural daylight hours of the season (i.e., 15.5:8.5, light/dark). Individuals continued to be provided with both food and water ad libitum until the start of their exposure session. For the exposure sessions, males and females were equally distributed across five treatment groups, which differed in either particle size (50 or 100 nm particle aggregates, or no particles), or air temperature (5 or 25 °C, Figure 1A). Fifty individuals were euthanized right after exposure, and the remaining ten individuals were euthanized 2 weeks after (Figure 1C). For the two-week period, these individuals were transported back to the indoor aviaries. All individuals were weighed before and after their exposure session using a Pesola Spring scale (±0.1 g). Blood samples (50–100 μL) from the jugular vein were also collected 1 week prior to the start of the experiment and immediately after exposure (Methods Supporting Information).

### Choice of Model Particles

To mimic the physical particle characteristics of ambient ultrafine particles that are critical for the particle lung deposition, we used model particles that consisted of nonsoluble tin oxide (SnO_*x*_, 2 ≥ *x* ≥ 1) aggregates with comparable effective density to ambient particles.^60^ For hydrophobic particles, such as soot, the chemical particle composition does not affect deposition. SnO_*x*_ model particles were used because: (1) they could steadily be generated at high concentrations with well-defined, tunable sizes in the relevant interval during the entire exposure period (Figure S1), (2) Sn could be detected at low concentrations (∼0.005 mg·kg^–1^ tissue), and (3) the background Sn levels in the avian tissue of the study individuals was low (<0.01 mg·kg^–1^ tissue, Figure S2); hence, any increase in Sn levels in tissues was due to experimental exposures.

### Particle Generation

A spark discharge generator (SDG) was used to generate an aerosol consisting of air and SnO_*x*_ particles. More detailed information about the SDG principle has been thoroughly reported elsewhere.^61^ The resulting aerosol was produced at a flow rate of 2.5 L·min^–1^.

To obtain a quasi-monodisperse distribution, the generated particles from the SDG passed through a beta-emitting ^63^Ni neutralizer to obtain a bipolar charge distribution before entering a differential mobility analyzer (DMA,TSI 3081), which selects particles according to their electrical mobility diameter.^18^ The electrical mobility diameter (equivalent to mobility diameter) is directly related to the particle diffusion coefficient, irrespective of particle morphology, and is therefore the most appropriate size metric for describing diffusion-driven deposition in the respiratory tract.^39^ Accordingly, the size-dependent deposition efficiencies reported here are applicable to both spherical and nonspherical particles, including fractal aggregates, if particle size is given as mobility diameter. The mobility diameter is the standard sizing metric for submicrometre particles for ambient aerosols.

In our experiment, particles with an average electrical mobility diameter corresponding to singly charged particles of either 50 or 100 nm were selected for the exposure time, depending on the experimental treatment. Once the particles were generated by the SDG and passed through the DMA, the particle flow, containing only the size-selected particles, was connected to a flow-through respirometry circuit (Figure 1B). A smaller fraction of larger particles was also present (corresponding to doubly and triply charged particles) and accounted for in our particle deposition calculations. By number, the singly charged particles comprised 83 and 82% of the particles generated for each particle exposure type, respectively (i.e., 50 and 100 nm). The equivalent numbers in mass were 70 and 66% of the 50 and 100 nm particles, respectively (details Supporting Information, Table S1).

### Experimental Procedure

We carried out three 2 h exposure sessions per day for 10 consecutive days in August 2022. In each exposure session, a pair of individuals was placed in separate hermetic glass respirometer chambers (1.2 L), which in turn were placed inside a climate test chamber (Weiss Technik WK3 C180, Reiskirchen, Germany). Temperature inside the respirometer chambers was measured in approximately 50% of exposures using thermocouples (36-gauge, Type T, copper/constantan). Birds were placed in the chamber 1 h prior to exposure to allow acclimation to both the chamber environment (ventilated with ambient air) and the experimental air temperature.

During the acclimatization hour, airflow passed through the SDG to the respirometer chamber without particle generation. The DMA voltage was set to a mobility diameter corresponding to 0 nm to ensure particle-free air. The temperature in the climate chamber was set to the correct experimental temperature (either 5 or 25 °C). To ensure a stable particle output at the start of particle exposure, the particle generation in the SDG was turned on toward the end of the acclimatization hour, but without allowing particles to pass into the chambers until the start of exposure. For the control treatment groups, the settings were kept the same between the acclimatization hour and the 2 h exposure (i.e., no particle generation and particle size in the DMA set to 0 nm).

Stable particle generation throughout the exposure hours was secured by continuous online monitoring of the particle number size distribution using an scanning mobility particle sizer (SMPS, TSI model 3082), composed by a DMA (TSI 3081) and a Condensation Particle Counter (CPC, TSI 3775). The SMPS was run using an aerosol to sheet flow of 0.3:3 L·min^–1^. If needed, the particle generation parameters were adjusted to generate a stable particle output. All individuals completed their exposure sessions without complications.

After the exposure session, particle generation in the SDG was turned off, and the desired particle size in the DMA was set back to 0 nm. We removed the individuals from their respective respirometer chambers and transported them to the dissection room within 5 min of the end of exposure. Lungs, heart and liver were carefully removed, weighed on a digital scale (±0.001 g, Methods Supporting Information) and kept frozen at −80 °C for later analyses.

### Measurement of Metabolic Rate

We measured the flow of dry air passing through the respirometer chambers with an FB-8 mass flow meter (Sable Systems International, SSI; Las Vegas, NV, USA). An empty respirometry chamber was used to record baseline values of gas concentrations. Mean air flow was 510.40 ± 79.60 mL·min^–1^ (mean ± SD; at standard temperature and pressure, dry, STPD). For the gas measurements, we subsampled 138.80 ± 8.64 mL·min^–1^ (STPD) from the main flow using a SS4 subsampler (SSI). Oxygen levels in the air flow were measured with a FC-10 analyzer (SSI), water vapor pressure with a RH-300 analyzer (SSI), and carbon dioxide levels with CA-10 analyzer (SSI). Air from each respirometer chamber and the control chamber was sampled sequentially in 7.5 min measurement intervals until the end of the 2 h exposure. The oxygen meter was span calibrated to 20.95% using dry room air every second day. The carbon dioxide meter was zero and span calibrated by using 100% N_2_ and 0.5% CO_2_, respectively, before the start of the experiment. The RH-300 was zero calibrated using dry air (drierite), and span calibrated by exploiting the dilution of oxygen by water vapor in moist air (following ref 62), also immediately before the experiment started.

### Assessment of Particle Morphology, Exposure Levels and Size Distributions

To investigate primary particle size and morphology, both 50 and 100 nm particles were deposited on lacey carbon copper grids and analyzed using a transmission electron microscope (TEM, JEOL 3000F). The average primary particle size was determined by measuring 110 primary particles from 20 agglomerates (evenly split between the 50 and 100 nm samples; details in Supporting Information Methods, Figure S1). Using Fast Fourier transform analysis of high-resolution TEM-images, lattice distances corresponding to both SnO and SnO_2_ could be identified, confirming their presence. Recent work including surface analysis of similar particles revealed that the surface is dominated by SnO_2_.^63^

We determined particle mass concentrations (in μg·m^–3^) for each exposure session by collecting particles on a cellulose filter and analyzing Sn content by inductively coupled plasma mass spectrometry (ICP–MS, ALS Scandinavia AB Laboratory Group). To calculate exposure levels (i.e., the mass concentration in the air), the obtained mass of Sn particles was divided by the air volume sampled through the filter.

The exposure level was measured at each session to know the exposure of each individual and was later used to calculate the deposition rate (DR) and deposition efficiency (DE) from the deposited dose determined for that individual. This way we accounted for any variation in concentration between the different exposure sessions. The mass size distribution was determined from the measured number size distribution by the SMPS, assuming an effective density decreasing with size, as described earlier,^60,64^ and a fractal dimension of 2.2, also from earlier measurements.

The DMA selects particles of one electrical mobility diameter. While most particles are carrying a single charge, a fraction of particles passing through the DMA is known to have multiple charges.^65^ Particles of multiple charges passing the DMA will have a larger mobility size than the singly charged particles. In our setup, the number of doubly and triply charged particles were determined by the SMPS after recharging the particles with a neutralizer, and the average particle size distribution on each exposure session was fitted with log–normal distributions. Details and distributions are presented in Supporting Information (Table S1), in which we show the stability in particle size distribution and the fraction of singly, doubly, and triply charged particles, both with respect to number and mass concentrations. This information was used in our later calculations of the size dependent dose rate and deposited fraction.

The average mass level in the air during exposure, and the mass of singly charged particles (combining measured mass levels with data on mass fraction of singly charged particles), are given in Table 1. Number size distributions are shown in Figure 2B.

### Deposited Particle Dose in the Lungs

We quantified deposited particle dose in the lungs of all exposed individuals (*n* = 60) using ICP–MS. Each measured individual belonged to one of the five treatment groups (Figure 1A): 50 nm particle aggregates at 25 °C (*n* = 10), 100 nm particle aggregates at 25 °C (*n* = 20), particle-free air at 25 °C (*n* = 10), 100 nm particle aggregates at 5 °C (*n* = 10), or particle-free air at 5 °C (*n* = 10). The two treatments with particle-free air were used as controls for their respective experimental temperatures (5 and 25 °C).

### Particle Translocation to Circulation or to Extrapulmonary Organs

Using ICP–MS, we investigated whether particles had reached circulation or extrapulmonary organs (liver and heart) in two subsets of individuals (subset 1: *n*_circulation_ = 14, including 5 individuals exposed to 50 nm particles at 25 °C, 5 individuals exposed to 100 nm particles at 25 °C, and 4 individuals at 5 °C; subset 2: *n*_extrapulmonary organs_ = 14, with all individuals in this subset exposed to 100 nm particles at 25 °C). To determine whether inhaled particles had moved to other parts of the body over time, the subset where extrapulmonary organs were measured included both individuals euthanized right after exposure (*n* = 4) and 2 weeks after (*n* = 10).

### Deposited Dose Rate (DR) in the Lungs

We determined “deposited dose rate (DR)”, defined as deposited dose per hour and per particle mass concentration in the inhaled air (per μg·m^–3^). DR is calculated as:

3 $$DR=\frac{deposited\;dose\;\left(\mu g\right)}{\mathrm{exposure}\;time\;\left(h\right)\times particle\;concentration\;in\;air\;\left(\mu g\cdot m^{-3}\right)}$$

This measure is independent of whether the exposure levels vary slightly between the exposures of different individuals and can be used to determine the dose of particles to zebra finches, in any setting, if the particle concentration in air is known.

To calculate the DR of 50 and 100 nm particles in the lungs, we also corrected for the fraction of doubly charged particles determined at each exposure. This correction is needed as DR is expected to vary with particle size. We implemented this correction using an iterative approach. First, we assumed a true monodisperse particle distribution for the respective treatment (50 or 100 nm; Figure 2C) and calculated the deposition rates accordingly. We then linearly interpolated these estimates of the deposition rates for 50 and 100 nm particles to determine the DR of doubly and triply charged particles based on their measured size and relative concentration. For particles larger than 100 nm, we estimated deposition rates of doubly charged particles by extrapolating from known size-dependent decreases in diffusional deposition efficiency in a tube.^18^ Knowing the fractions of singly, doubly and triply charged particles, we then recalculated DRs. This procedure was iterated up to 10 times as no differences were observed between the ninth and the 10th iterations. Although we identified this approach as the most accurate, the correction on DR using the iterative approach was only 4% for 50 nm particles and 10% for 100 nm particles.

### Estimated Deposition Efficiency (DE) in the Lungs

We obtained DE by dividing an individual’s DR by the minute ventilation rate converted to hourly volume ventilation rate (*V*_e_, in m^3^·h^–1^). DE is the probability that a particle, once inhaled, will be deposited in the lung, and is given as %. DE can be applied to estimate the dose of particles in different bird species based on the concentration of particles in the air (in μg·m^–3^) and their minute volume ventilation (in m^3^·min^–1^). DE is defined as:

4 $$DE=\frac{deposited\;dose\;\left(\mu g\right)}{particle\;concentration\;in\;air\;\left(\mu g\cdot m^{-3}\right)\times time\left(h\right)\times V_{e}\left(m^{3}\cdot h^{-1}\right)}\times 100=\frac{DR\;\left(\mu g{\cdot h}^{-1}\;per\;\mu g\cdot m^{-3}\;in\;air\right)}{V_{e}\left(m^{3}\cdot h^{-1}\right)}\times 100$$

where *V*_e_ is minute volume ventilation rate converted to hourly volume ventilation rate.

To calculate minute ventilation, we used the measured oxygen consumption levels of each individual during exposure (in m^3^·min^–1^, STPD) and the relationship between oxygen consumption (in m^3^·min^–1^, STPD) and minute ventilation (in m^3^·min^–1^, at body temperature) in zebra finches reported in the literature (ref 66, as reported in ref 67). Volume flows were corrected for the temperature difference between standard conditions (STPD; 0 °C) and body temperature. To account for biological variation between individuals, we used the mean *V*_e_ of the 2 h exposures of all birds within each temperature, as oxygen consumption was higher in the cold condition (as expected). Note that any error in the estimation of *V*_e_ scales linearly to DE.

### Simplified Theoretical Calculations of Particle Deposition

We performed first-order calculations of particle deposition based on fundamental aerosol physics and an idealized representation of the avian respiratory system. Rather than developing a comprehensive deposition model, we tested whether diffusion alone could reproduce the experimentally observed size-dependent deposition pattern and whether the results are consistent with deposition occurring in the trachea and air sacs.

Deposition due to gravitational settling, Brownian diffusion, and inertial impaction at airway junctions was estimated by representing the respiratory tract as a simplified flow-through tube system, and using the equations described in^41,68^ for particle deposition. In the calculations we assumed laminar flows.

### Particle Clearance from the Lungs

To estimate particle clearance in the lungs of individuals euthanized 2 weeks after exposure, we first quantified the remaining deposited dose in their lungs (using ICP–MS). Additionally, we had to estimate the initial deposited particle mass. This was done by multiplying the mean DR of individuals euthanized right after exposure (calculated from data from individuals exposed to the same particle size and temperature as those used for the clearance estimation) with the measured exposure levels of the clearance individuals and the exposure time (see eq 3). Clearance was assessed for 100 nm particles and included the doubly and triply charged particles.

### Calculation of Metabolic Rate

All data were recorded using ExpeData software (version 1.9.27, Sable Systems). Oxygen consumption (in mL·min^–1^) was calculated after mathematically “drying” the gas using eq 8.7 by ref 62, based on the two most stable min in each 7.5 min recording. We then calculated metabolic rate (in W) by assuming an energy equivalence of 20 J per 1 mL of oxygen.^69^ We obtained four direct measurements of metabolic rate at regular intervals throughout the 2 h exposure period. We also recorded two additional measurements per individual during the acclimatization hour, which we averaged and used as their baseline value. Due to a technical error, one individual had only a single measurement during the acclimatization hour, so we used that value instead as their acclimatization estimate.

### Oxidative Status Biomarkers in Plasma

We measured the nonenzymatic antioxidant capacity (OXY adsorbent test kit; Diacron International, Grosseto, Italy) and oxidative damage levels (d-ROMs test kit; Diacron International, Grosseto, Italy) in plasma before and after exposure. Antioxidant levels were quantified by measuring the capacity of the antioxidant barrier of a sample to combat the action of an oxidant solution (hypochlorous acid, HClO, Methods Supporting Information). Oxidative damage was estimated as the concentration of Reactive Oxygen Metabolites (ROMs), a class of compounds (mostly hydroperoxides, ROOH) produced when oxidative stress occurs to lipids, proteins and nucleic acids (Methods Supporting Information). For each assay, samples were run in duplicates (including the standard curve dilutions for the calculations, and the water blank), and treatments were balanced across plates, with pre- and postexposure samples from the same individual run together on the same plate (except for 8 samples in the ROMs assay, which needed to be rerun in a different plate).

### Statistical Analyses

We conducted all analyses in R, version 4.2.1.^70^ An alpha level of 0.05 (i.e., *p* < 0.05) was used to determine statistical significance. Models were produced using the package “lme4”.^71^ Data outliers were identified both visually and using Rosner’s test (Generalized Extreme Studentized deviate) in the “EnvStats” package.^72^ Two outliers were detected in the model testing the effect of temperature on DE, but they were kept in the analysis because they reflected true measurements and did not significantly affect the model output. Normality of model residuals and model outliers were evaluated for all models. For linear mixed models, residual normality was identified using the package “DHARMa”.^73^ Estimates were calculated using ordinary least-squares (OLS) in linear models, and the restricted maximum likelihood method (REML) in linear mixed models. For the latter, denominator degrees of freedom were calculated using Kenward-Roger’s approximation.

Due to our unbalanced design, we ran two separate linear models (LMs) to test the effect of either particle size (“50 nm”, “100 nm”) or temperature (“5 °C, “25 °C”) on DR (μg·h^–1^ per μg·m^–3^ in air) and DE (%). These LMs had the same total sample size each (*n* = 20), and control treatments were excluded. Body mass (average from before and after exposure values, in g) was included as a covariate, and sex (“Male”, “Female”) as a fixed effect. Other variables in our experiment were excluded from the models due to different reasons (Supporting Information Methods).

To test for a significant difference between the estimated initial deposited particle mass and the measured remaining mass in individuals euthanized 2 weeks after exposure, we used a Student’s *t*-test.

To analyze the effect of particle exposure on metabolic rate, we ran two different linear mixed models (LMMs), one per temperature treatment (i.e., 5 or 25 °C). In both models, the interaction between “Measurement time point (from 1 to 5) and “Treatment” was initially included. Measurement time point 1 corresponded to the average metabolic rate during the acclimatization hour (baseline value), and 2–5 referred to the measurements taken twice an hour from the start to the end of the 2 h exposure. “Treatment” consisted of different groups depending on the temperature model, including the correspondent control group per temperature (Table S6). This interaction term was included to test whether any potential change in metabolic rate between baseline levels and particle exposure levels was different between treatments, but we dropped it from the final models due to nonsignificance (see Results). To account for repeated measurements of the same individual, “Bird ID” was added as a random factor.

To assess the effect of particle size and temperature treatments on nonenzymatic antioxidant capacity and oxidative damage, we built two separate LMMs. Our response variables were the levels of antioxidants and oxidative damage after exposure, respectively. We included all five treatments under “Treatment” (Table S7). “Sex” was also included as a fixed effect. Pre-exposure levels of either antioxidant capacity or oxidative damage, as well as average body mass, were added as covariates. Only individuals with samples from both before and after exposure were included in the models (Table S7). To account for variation between assay plates, “Plate number” (1−6) was added as a random factor.

## Supplementary Material







## Data Availability

All our study data is included as Supporting Information. R code is available upon request.

## Notes

Data set S1 (separate from SI file): Particle size distributions, primary particle sizes, daily mass distributions and particle number and mass concentrations during experimental exposures (XLSX)
Data set S2 (separate from SI file): Particle dose, deposition rate, deposition efficiency, particle clearance, metabolic rate and oxidative status measurements in adult zebra finches (XLSX)
Methods—extended text. Figures S1–S2. Tables S1–S7 and SI References (PDF)
