# Supplementary material for: Ultrafine Airborne Particle Deposition and Clearance in an Avian Lung under Ecologically Relevant Conditions
Source: Environ Sci Technol. 2026 Aug 24;60(35):24591–603. doi: 10.1021/acs.est.6c05957 (PMC13564425; doi:10.1021/acs.est.6c05957)
Supplement: Supplementary file 1 [file es6c05957_si_003.pdf]

Supporting Information

Ultrafine airborne particle deposition and clearance in an avian lung under ecologically relevant conditions

Susana Garcia Dominguez[1], Linnéa Jönsson[2,3], Andreas Nord[1,4], Calle Preger[3,5,6], Maria E. Messing[2,3,7], Caroline Isaksson[1†*], Jenny Rissler[3,5†*]

[1] Department of Biology, Lund University, 223 62 Lund, Sweden

[2] Solid State Physics, Department of Physics, Lund University, 221 00 Lund, Sweden

[3] NanoLund, Lund University, 221 00 Lund, Sweden

[4] Swedish Centre for Impacts of Climate Extremes (climes), Lund University, 221 00 Lund, Sweden

[5] Ergonomics and Aerosol Technology, Department of Design Sciences, Lund University, 221 00 Lund, Sweden

[6] MAX IV Laboratory, Lund University, Box 118, Lund 221 00, Sweden

[7] Quantum Device Laboratory, Department of Microtechnology and Nanoscience, Chalmers University of Technology, 412 96 Gothenburg, Sweden

†Shared last authorship.

*Caroline Isaksson, Jenny Rissler.

Email: caroline.isaksson@biol.lu.se; jenny.rissler@design.lth.se

This Supporting Information (pages S1-S13) includes:

Supporting text: Methods - extended (pages S2-S4)

Figures S1-S2 (pages S5-S6)

Tables S1-S7 (pages S7-S14)

Dataset 1 (separate file, summary of contents on page S13)

Dataset 2 (separate file, summary of contents on page S13)

SI References (page S13)

Supporting Information Text

Methods – extended.

*Particle size and morphology - extended***.** The primary particle diameter was estimated from TEM-images of 10 agglomerate particles of each sample. As the primary particles appear to be partially merged with their particle neighbours, the analysis of their individual diameter is not straightforward. For this analysis, we chose to measure the diameters of the crystalline primary particles from the TEM-Images, to have an estimate of where the edge of the primary particle is. Furthermore, the primary particles are not always spherical in shape, which is why the diameter was estimated as the average of a measurement in the horizontal and the vertical direction. For each sample, the diameter of 55 primary particles was measured (Fig. S1).

Accounting for the presence of doubly and triply charged particles in the deposited dose. A quasi-monodisperse aerosol was produced by directing the particles generated by the SDG through a ^{63}Ni β-source, which imposed a bipolar charge distribution. The aerosol then entered a DMA (TSI 3081), which selects particles according to their electrical mobility diameter. For each experimental condition, the DMA was set to transmit particles with a mobility corresponding to singly charged 50-nm or 100-nm particles. Because a bipolar charge distribution inevitably contains particles with multiple charges, the DMA also transmits larger particles that attain the same mobility through double or triple charging. To determine the true size distribution and determine the number of particles associated with each charge state, we re-neutralized the aerosol and measured the resulting number-size distribution using the SMPS system (described in main paper). The measurements produced size fractions consistent with theoretical predictions. The proportions and corresponding sizes of singly, doubly, and triply charged particles are summarized in Table S1.

Since deposition efficiency depends on particle size, these multiple charge states must be considered when estimating deposited dose, dose rate, and deposition behaviour for the nominal 50 and 100 nm aerosols. The information on multiply charged particles was also used to estimate deposition for the corresponding larger physical diameters (up to 200 nm), shown in Fig. 2C. To do so, an iterative procedure was applied. We first assumed a perfectly monodisperse aerosol and calculated deposition for the nominal sizes. These initial deposition values were then linearly interpolated between 50 and 100 nm to estimate deposition for the doubly and triply charged fractions based on their measured sizes and concentrations. Particles larger than 100 nm required an initial estimate using the known dependence of diffusional deposition efficiency in tubes on particle size (1). Using these values, deposition rates for all charge states were recalculated, and the procedure was repeated ten times. No changes were observed between the ninth and tenth

iterations, indicating convergence. This correction caused a modest increase in the estimated dose: approximately +4% for the 50-nm aerosol and +10% for the 100-nm aerosol.

Experimental procedure- extended. For the pre-exposure biomarker levels, a blood sample from the jugular vein (~50 μL) was taken from each individual before release to the indoor aviary (~1 week before exposure). To separate plasma from red blood cells, samples were centrifuged for 10 minutes at 4000 rpm and kept on ice for ~3 hours before they could be stored in a -80°C freezer. For the post-exposure sample, the same sampling procedure (but higher blood volume sampled, ~100 μL) was used.

The first exposure session started at 08:22 am ± 22 min (mean ± SD), whereas the last session started at 15:05 pm ± 48 min. Treatment groups were spread evenly throughout exposure session slots during the experimental period (i.e., session 1, 2 or 3), but not within each day (i.e., the three session exposures within a day could all be part of the same treatment group). Inside the respirometer chamber, the individuals were able to move freely without flying.

To avoid particle transmission between the exposed individuals and the unexposed ones, we always handled the exposed individuals with nitrile gloves. We also used single-use paper bags for transport after exposure and thoroughly cleaned the respirometer chambers using cleanroom cloths and isopropanol. Individuals were euthanized by decapitation immediately after the post-exposure blood sampling. To minimize particle contamination, we used new gloves for organ removal, and tools free of tin traces throughout the experiment.

Oxidative status biomarkers – extended. To obtain the levels of non-enzymatic antioxidant capacity (in mM of neutralized hypochlorous acid, HClO) of each sample, we diluted 2 μL of plasma with 198 μL of double-distilled water (1:100 ratio) in a 96-well microplate, and mixed 2 μL of the diluted sample with 200 μL of the oxidant solution (1:100 ratio). After incubating the samples at 37°C for 10 minutes (with shaking at 500 rpm), we added 2 μL of a chromogenic mixture containing an alkyl aromatic amine ($A\text{-}NH_2$). The leftover oxidant solution not neutralized by the barrier reacted with the amine. Once the chromogenic mixture was added in each well, absorbance was measured immediately at a wavelength of 490 nm in a microplate reader (FLUOstar OMEGA, BMG LABTECH). The inter-assay coefficient of variation (CV) between plates was 10.23% (calculated from the average of the standard curve slopes from the plates used, n = 3), whereas the intra-assay CV between sample duplicates was 1.04% (calculated from the average of all CVs from all sample duplicates, n = 113, Table S6).

To measure oxidative damage (in mM of H_2O_2) of each sample, we diluted 4 μL of plasma with an acidic buffer solution, which contained an acetate buffer (pH 4.8) and a chromogenic mixture with *N,N*-diethyl-p-phenylenediaminein a 1:100 ratio. The acetate buffer helped release iron from proteins in the plasma, which triggered the generation of radicals that react with the amine. After incubating the samples in 96-well plates at 37°C for 75 minutes, the samples were centrifuged in a plate centrifuge for two minutes at 5700 r.p.m. Absorbance at a wavelength of 490 nm was measured using a microplate reader (FLUOstar OMEGA, BMG LABTECH). The inter-assay coefficient of variation (CV) between plates was 5.54% (calculated from the average of the standard curve slopes from the plates used, n = 5), whereas the intra-assay CV between sample duplicates was 4.71% (calculated from the average of all CVs from all sample duplicates, n = 94, Table S6).

Statistical analyses – omission of variables. Individual age was not included in the models as the majority of individuals in each treatment group were four years old. Individual body mass (i.e., average mass measurements before and after exposure) positively correlated with lung weight ($r_s = 0.427$, $p < 0.001$, n = 60). Therefore, we only included body mass

Figures

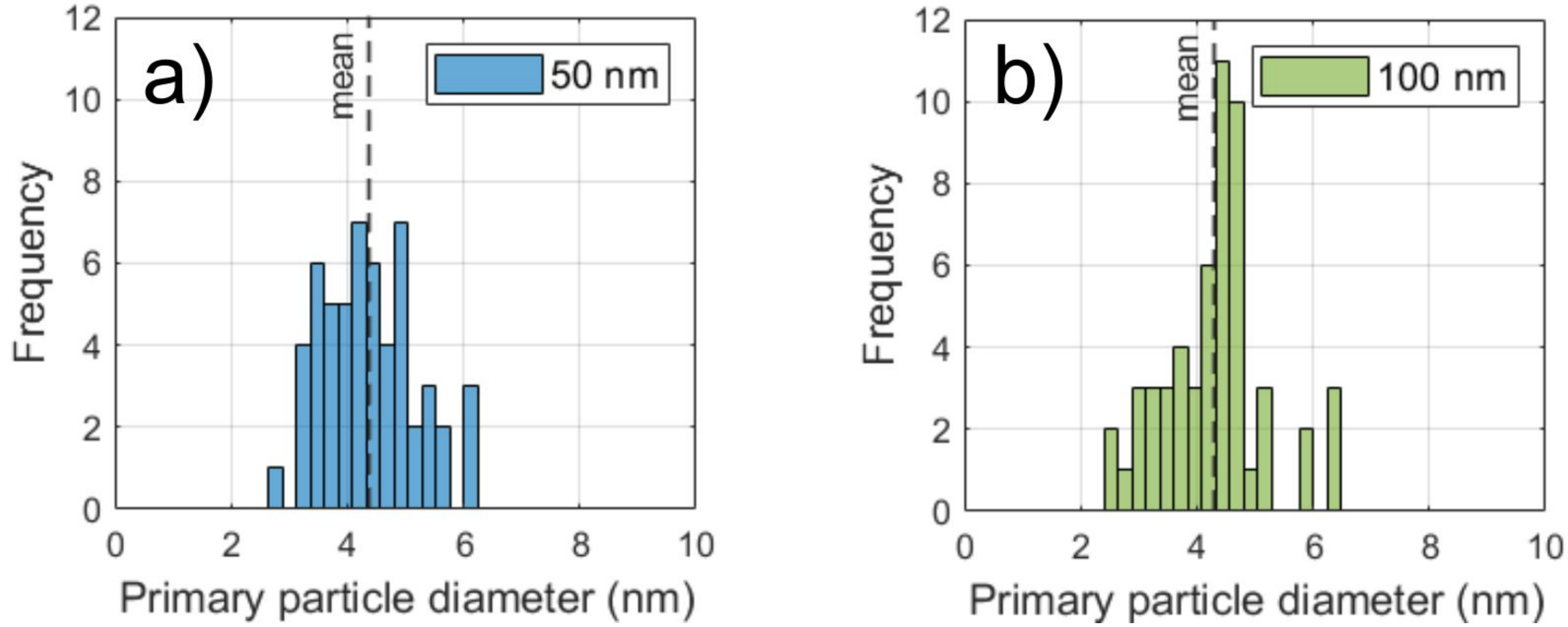

Fig. S1. Size distributions of the primary particles for both 50 nm (a) and 100 nm (b) particles.

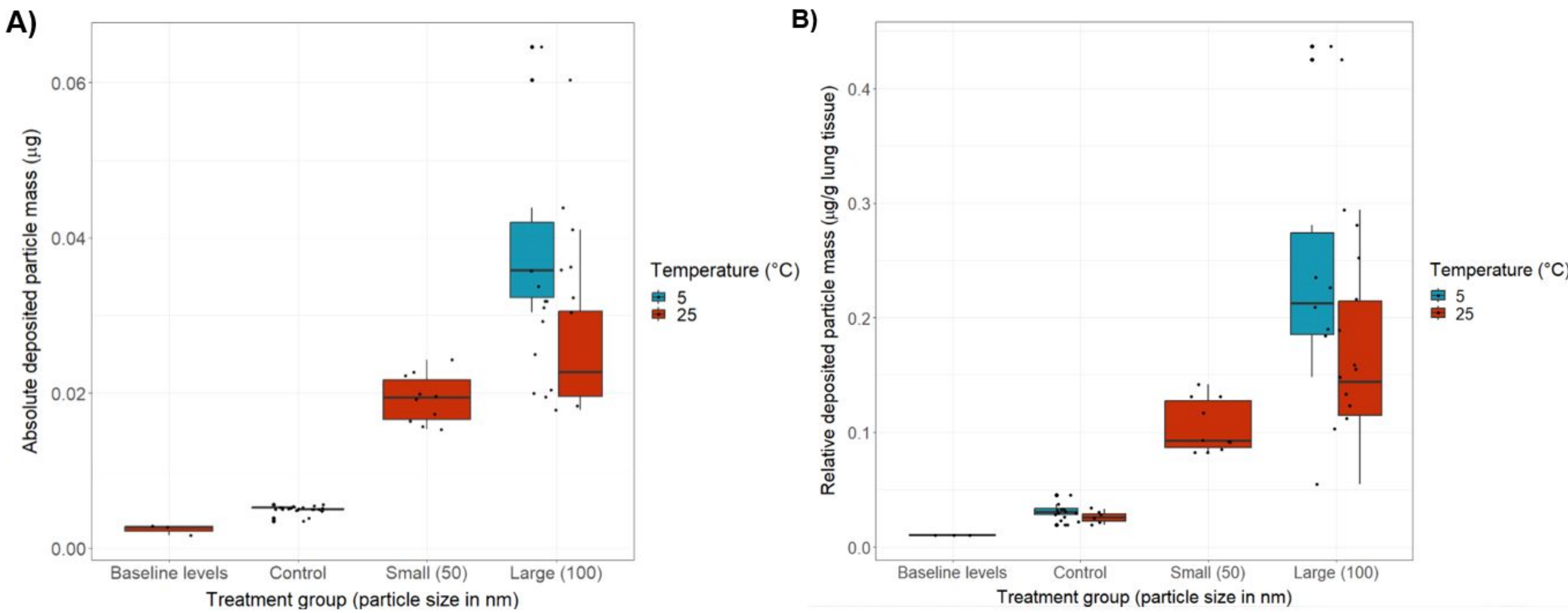

Fig. S2. Deposited particle mass of Sn in the lungs of adult zebra finches exposed to different treatment groups. A) Absolute mass shown in µg, and B) relative mass shown in µg·g^{-1} lung tissue. Individuals are grouped by treatment, depending on particle size: 'Small (50)' or 'Large (100)' (particles with mobility diameter of 50 or 100 nm, respectively), and air temperature (either 5 or 25 °C). 'Control' refers to individuals exposed to no particles. 'Baseline levels' refer to Sn levels measured in three individuals from the population that did not participate in the particle exposures. Raw data shown as black dots, and data outliers shown as bold black dots.

Tables

Table S1. Overview of particle characteristics data for fitted mass size distributions. For mass size distributions, the number fractions of singly charged particles were 70 and 66%, for each particle size treatment, respectively (i.e., 50 and 100 nm, both at 25 °C). The corresponding numbers for the number size distributions were 83 and 82%.

		Singly charged	Doubly charged	Triply charged
Small (50 nm)	Dp (nm)	49.6 ± 0.8	70.8 ± 1.0	87.2 ± 1.1
	σ_g	1.08 ± 0.00	1.08 ± 0.00	1.08 ± 0.00
	%	69.8 ± 0.6	23.4 ± 0.5	6.8 ± 0.2
Large (100 nm)	Dp (nm)	103.7 ± 1.5	153.4 ± 1.8	196.2 ± 2.1
	σ_g	1.09 ± 0.00	1.09 ± 0.00	1.09 ± 0.00
	%	65.8 ± 1.9	24.2 ± 1.1	10.0 ± 1.2

Numbers are based on fitting log-normal distributions to the peaks in the mass-size distributions. 'Dp' refers to the particle geometric mean diameter, and 'σ_g' to the geometric standard deviation of the distribution (broadening). Numbers given as % correspond to the particle mass respective to the total mass fraction of the distribution. Mean ± 1 SD shown, where SD represents the standard deviation between exposures.

Table S2. Descriptive statistics for 1) total particle deposited dose in lungs, and 2) dose of only singly charged particles, in adult zebra finches.

TOTAL PARTICLE MASS

**Absolute mass (μg)**			
	**Range**	**Mean ± SE**	**Median ± IQR**
**Clearance period 0 days**			
Large, 5 °C	0.030-0.065	0.040 ± 0.004	0.036 ± 0.010
Large, 25 °C	0.018-0.041	0.025 ± 0.002	0.023 ± 0.011
Small, 25 °C	0.015-0.024	0.019 ± 0.001	0.019 ± 0.005
Control	0.003-0.006	0.005 ± (<0.001)	0.005 ± (<0.001)
**Clearance period 14 days**			
Large, 25 °C	0.013-0.033	0.019 ± 0.002	0.018 ± 0.004
**Relative mass per lung mass (μg·g^{-1})**			
	**Range**	**Mean ± SE**	**Median ± IQR**
**Clearance period 0 days**			
Large, 5 °C	0.148-0.437	0.250 ± 0.033	0.213 ± 0.089
Large, 25 °C	0.055-0.294	0.163 ± 0.023	0.144 ± 0.010
Small, 25 °C	0.082-0.142	0.105 ± 0.007	0.092 ± 0.041
Control	0.019-0.045	0.028 ± 0.002	0.029 ± 0.010
**Clearance period 14 days**			
Large, 25 °C	0.063-0.304	0.123 ± 0.025	0.087 ± 0.063

SINGLY CHARGED PARTICLE MASS

**Absolute mass (μg)**			
	**Range**	**Mean ± SE**	**Median ± IQR**
**Clearance period 0 days**			
Large, 5 °C	0.022-0.043	0.029 ± 0.002	0.026 ± 0.011
Large, 25 °C	0.014-0.028	0.019 ± 0.002	0.017 ± 0.009
Small, 25 °C	0.011-0.018	0.014 ± 0.001	0.014 ± 0.004
Control	N.A.	N.A.	N.A.
**Clearance period 14 days**			
Large, 25 °C	N.A.	N.A.	N.A.
**Relative mass per lung mass (μg·g^{-1})**			
	**Range**	**Mean ± SE**	**Median ± IQR**
**Clearance period 0 days**			
Large, 5 °C	0.107-0.295	0.176 ± 0.021	0.155 ± 0.096
Large, 25 °C	0.041-0.215	0.119 ± 0.016	0.108 ± 0.071
Small, 25 °C	0.060-0.103	0.076 ± 0.005	0.068 ± 0.033
Control	N.A.	N.A.	N.A.
**Clearance period 14 days**			
Large, 25 °C	N.A.	N.A.	N.A.

Size refers to the mobility diameter of the particles individuals were exposed to, 50 nm (Small), or 100 nm (Large). Temperature refers to the air temperature, cold (5°C) or mildly warm (25°C). 'Clearance period 0 days' includes those individuals euthanized right after exposure, whereas 'Clearance period 14 days' consists of those euthanized two weeks after. Ranges, means ± standard error (SE), and medians ± interquartile range (IQR) are shown for absolute deposited particle mass and relative deposited particle mass to lung mass. Values are shown per treatment. 'N.A.' = not applicable. N = 10 for all treatment groups except for 'Control', N = 20.

Table S3. Statistical output of linear models exploring treatment effects on deposited dose rate and estimated particle deposition efficiency in zebra finches.

Response variable	Predictor	Estimate[1] ± SE[1]	SS[1]	df	F	P
Dose rate $\cdot 10^{-4}$ [μg·h^{-1} per μg·m^{-3}]	Intercept	1.24 ± 0.31				
	**Particle size (100 nm)**	**-0.67 ± 0.09**	**2.51**	**1**	**65.86**	**<0.001**
	Sex (male)	-0.01 ± 0.09	<0.01	1	0.01	0.94
	Body mass	0.02 ± 0.02	0.05	1	1.25	0.28
Dose rate $\cdot 10^{-4}$ [μg·h^{-1} per μg·m^{-3}]	Intercept	1.11 ± 0.19				
	**Temperature (25°C)**	**-0.51 ± 0.06**	**1.29**	**1**	**86.20**	**<0.001**
	Sex (male)	-(<0.01) ± 0.06	<0.01	1	0.00	0.96
	Body mass	0.02 ± 0.01	0.03	1	1.95	0.18
Estimated deposition efficiency	Intercept	8.50 ± 2.16				
	**Particle size (100 nm)**	**-4.63 ± 0.64**	**118.80**	**1**	**65.86**	**<0.001**
	Sex (male)	-0.05 ± 0.60	0.01	1	0.01	0.94
	Body mass	0.16 ± 0.14	2.25	1	1.25	0.28
Estimated deposition efficiency	Intercept	4.74 ± 1.03				
	Temperature (25°C)	-0.22 ± 0.29	0.25	1	0.59	0.45
	Sex (male)	-0.07 ± 0.29	0.01	1	0.01	0.91
	Body mass	0.11 ± 0.08	0.90	1	2.11	0.17

[1] Values are expressed in the order of 10^{-4} for Dose rate models.

'Particle size' indicates whether individuals were exposed to particles of 50 or 100 nm in mobility diameter. 'Body mass' (g) is the mean mass per individual from before and after exposure measurements. 'Sex' refers to either male or female. 'Temperature' refers to whether individuals were exposed to an ambient temperature of 25 °C or 5 °C. Estimates (with corresponding level shown in brackets) are expressed in μg·h^{-1} per μg·m^{-3} in air and in percentage, respectively, for dose rate and estimated deposition efficiency. N = 20 for each model. $P < 0.05$ = statistical significance, marked in bold.

Table S4. Statistical output of linear mixed models analysing treatment effects on metabolic rate in adult zebra finches.

Response variable	Predictor	Estimate ± SE	SS	df (num, den)	F	P
Metabolic rate (W, 5°C)	Intercept	0.22 ± 0.30				
	Treatment (100 nm)	-0.06 ± 0.06	0.01	1, 17	1.15	0.30
	**Measurement timepoint**	**0.02 ± (<0.01)**	**0.06**	**1, 79**	**12.32**	**<0.001**
	Body mass	0.03 ± 0.02	0.01	1, 17	2.16	0.16
	Random effect	Variance ± SD				
	Bird ID	0.02 ± 0.12				
	Residual	0.01 ± 0.07				
Metabolic rate (W, 25°C)	Intercept	0.33 ± 0.09				
	Treatment (100 nm)	-0.07 ± 0.03	0.02	2,36	2.22	0.12
	(50 nm)	-0.04 ± 0.04				
	Measurement timepoint	<0.001 ± (<0.01)	0.01	1,159	1.08	0.30
	Body mass	0.01 ± 0.01	0.01	1,36	1.81	0.18
	Random effect	Variance ± SD				
	Bird ID	0.01 ± 0.08				
	Residual	<0.01 ± 0.07				

Metabolic rate is measured in $J{\cdot}s^{-1}$ (W, Watts). ‘Treatment’ refers to individuals exposed to either particle-free air at 5 °C (control) or 100 nm particles at 5 °C in the cold temperature model, and to those exposed to either particle-free air at 25 °C (control), 100 nm particles at 25 °C, or 50 nm particles at 25 °C in the warm air temperature model. ‘Measurement timepoint’ indicates the moment in which the metabolic rate measurement was recorded (1-5, with 1 being the baseline value recorded in the acclimatization hour, and 2-5 being values recorded during exposure, twice an hour). ‘Body mass’ (g) is the mean mass per individual from before and after exposure measurements. ‘Sex’ refers to male or female. Estimates (with corresponding level shown in brackets) and their standard errors (SE), Sum of Squares (SS), degrees of freedom (df; num = numerator, den = denominator), F-statistics (F) and significance values (P) are reported. Variance and standard deviation (SD) are shown for the random effects and the model residuals. N = 20 for the cold temperature model, and N = 40 for model. Statistically significant effects (P < 0.05) marked in bold.

Table S5. Statistical output of linear mixed models investigating treatment effects on oxidative stress biomarkers in adult zebra finches.

Response variable	Predictor	Estimate ± SE	SS	df (num, den)	F	P
Non-enzymatic antioxidant capacity (OXY)	Intercept	88.12 ± 33.33				
	**Pre-exposure OXY**	**0.65 ± 0.10**	**28123.20**	**1, 44.85**	**38.46**	**<0.001**
	Treatment (control, 25 °C)	4.02 ± 12.72	866.20	4, 43.83	0.30	0.88
	(100 nm, 5 °C)	10.56 ± 12.60				
	(100 nm, 25 °C)	4.33 ± 11.33				
	(50 nm, 25 °C)	-2.85 ± 13.77				
	Body mass	-1.98 ± 1.91	778.70	1, 43.34	1.52	0.31
	Sex (male)	11.70 ± 7.77	1654.20	1, 43.10	1.24	0.14
	Random effect	Variance ± SD	N Groups			
	Plate number	48.43 ± 6.96	3			
	Residual	731.32 ± 27.04				
Oxidative damage (ROMs)	Intercept	-0.02 ± 0.27				
	**Pre-exposure ROMs**	**0.15 ± 0.04**	**0.72**	**1, 40.90**	**11.70**	**0.001**
	Treatment (control, 25 °C)	0.03 ± 0.13	0.45	4, 39.88	1.82	0.14
	(100 nm, 5 °C)	-0.01 ± 0.12				
	(100 nm, 25 °C)	0.19 ± 0.10				
	(50 nm, 25 °C)	0.26 ± 0.13				
	Body mass	0.04 ± 0.02	0.21	1, 37.47	3.33	0.08
	Sex (male)	0.12 ± 0.08	0.14	1, 39.41	2.25	0.14
	Random effect	Variance ± SD	N Groups			
	Plate number	<0.01 ± 0.03	6			
	Residual	0.06 ± 0.25				

OXY is measured in mM HClO neutralized, ROMs is measured in mM H_2O_2. 'Pre-exposure OXY' and 'Pre-exposure ROMs' indicate the individual levels of either OXY or ROMs before particle exposure, respectively. 'Treatment' refers to individuals exposed to either particle-free air at 5 °C or 25 °C (controls), 100 nm particles at 5 °C or 25 °C), or 50 nm particles at 25 °C. 'Body mass' (g) is the mean mass per individual from before and after exposure. 'Sex' refers to either male or female. 'Plate number' is the plate on which the assay was run. Estimates (with corresponding level shown in brackets) and their standard errors (SE), Sum of Squares (SS), degrees of freedom (df; num = numerator, den = denominator), F-statistics (F) and significance values (P) are reported. Variance and its standard deviation (SD) are shown for the random effects and the model residuals. For the random effects, the number of groups per effect is also reported. N_{OXY} = 53, N_{ROMs} = 49. Statistically significant effects (P < 0.05) marked in bold.

Table S6. Sample sizes for the analysis of the effect of particle exposure on metabolic rate in adult zebra finches.

Treatment	Cold temperature model	Warm temperature model
Large, 5 °C	10	NA
Large, 25 °C	NA	20
Small, 25 °C	NA	10
Control, 5 °C	10	NA
Control, 25 °C	NA	10
**Total individuals**	**20**	**40**

Size refers to the mobility diameter of the particles individuals were exposed to, either 50 nm (Small), 100 nm (Large), or particle-free air (Control). Each model corresponds to one air temperature treatment (cold, 5°C; warm 25°C).

Table S7. Sample sizes for the analysis of the effect of particle exposure on oxidative status biomarkers in adult zebra finches.

	**Non-enzymatic antioxidant capacity**	**Oxidative damage**
Treatment	Final N (experimental N)	Final N (experimental N)
Large, 5 °C	9 (10)	8 (10)
Large, 25 °C	16 (20)	16 (20)
Small, 25 °C	9 (10)	9 (10)
Control, 5 °C	10 (10)	9 (10)
Control, 25 °C	9 (10)	7 (10)
**Total individuals**	**53 (60)**	**49 (60)**

Size refers to the mobility diameter of the particles individuals were exposed to, which was either 50 nm (Small), 100 nm (Large), or particle-free air (Control). Temperature refers to the air temperature, either 5°C or 25°C. 'Final N are the sample sizes used for the statistical analyses, which only include individuals with both pre- and post- exposure samples. 'Experimental N' (in brackets) includes all individuals in the experimental treatment group.

Dataset S1 (separate file)

Particle size distributions, primary particle sizes, daily mass distributions and particle number and mass concentrations during experimental exposures.

Dataset S2 (separate file)

Particle dose, deposition rate, deposition efficiency, particle clearance, metabolic rate and oxidative status measurements in adult zebra finches.

SI References

1. W. C. Hinds, Aerosol Technology: Properties, Behavior, and Measurement of Airborne Particles (Wiley Interscience, 1999).
